# Clinical Characteristics of Tuberculosis-Associated Immune Reconstitution Inflammatory Syndrome in North Indian Population of HIV/AIDS Patients Receiving HAART

**DOI:** 10.1155/2011/239021

**Published:** 2010-12-01

**Authors:** Suman Karmakar, Surendra K. Sharma, Richa Vashishtha, Abhishek Sharma, Sanjay Ranjan, Deepak Gupta, Vishnubhatla Sreenivas, Sanjeev Sinha, Ashutosh Biswas, Vinay Gulati

**Affiliations:** ^1^Department of Medicine, All India Institute of Medical Sciences, New Delhi 110029, India; ^2^Division of Pulmonary, Critical Care, & Sleep Medicine, Department of Medicine, All India Institute of Medical Sciences, Ansari Nagar, New Delhi 110029, India; ^3^University of Medicine, Pleven, Bulgaria; ^4^Department of Biostatistics, All India Institute of Medical Sciences, New Delhi 110029, India

## Abstract

*Background & Objective*. IRIS is an important complication that occurs during management of HIV-TB coinfection and it poses difficulty in diagnosis. Previous studies have reported variable incidence of IRIS. The present study was undertaken to describe the pattern of TB-associated IRIS using recently proposed consensus case-definitions for TB-IRIS for its use in resource-limited settings. *Methods*. A prospective analysis of ART-naïve adults started on HAART from November, 2008 to May, 2010 was done in a tertiary care hospital in north India. A total 224 patients divided into two groups, one with HIV-TB and the other with HIV alone, were followedup for a minimum period of 3 months. The diagnosis of TB was categorised as ‘‘definitive” and ‘‘probable”. *Results*. Out of a total of 224 patients, 203 completed followup. Paradoxical TB-IRIS occurred in 5 of 123 (4%) HIV-TB patients while 6 of 80 (7.5%) HIV patients developed ART-associated TB. A reduction in plasma viral load was significantly (*P* = .016) associated with paradoxical TB-IRIS. No identifiable risk factors were associated with the development of ART-associated TB. *Conclusion*. The consensus case-definitions are useful tools in the diagnosis of TB-associated IRIS. High index of clinical suspicion is required for an early diagnosis.

## 1. Introduction

Human immunodeficiency virus/acquired immunodeficiency syndrome (HIV/AIDS) and tuberculosis (TB) are leading causes of morbidity and mortality worldwide [1]. The incidence of TB is highest among patients with advanced HIV disease [2]. A majority of the global burden of HIV-associated TB lies in resource-constrained countries [1]. Antiretroviral treatment (ART) has been made widely available in these areas over last several years now. However, many patients in these countries start ART at a late stage when they have advanced HIV/AIDS [3]. The beneficial effects of ART result from gradual restoration of pathogen-specific immune responses mediated by suppressed HIV-1 replication and increased CD4 cell count [4]. The fact that many patients accessing ART are already receiving treatment for TB presents a major clinical challenge due to the complexities involved in the concurrent management of these two coinfections [5–8]. In addition to high pill burden, drug cotoxicity and pharmacokinetic drug interactions, “immune reconstitution inflammatory syndrome” (IRIS) also called “immune reconstitution syndrome” (IRS) has been a major problem. The syndrome is usually a consequence of exaggerated activation of the immune system against persistent antigen (paradoxical IRIS) or viable pathogens (unmasking IRIS), but it can also develop as progression of proliferative disease in patients with cancers [9]. IRS is associated with certain infectious (e.g., *mycobacteria, varicella zoster, cytomegalovirus*) and noninfectious (autoimmune or neoplastic) conditions. ART-induced IRS includes either a paradoxical worsening of treated opportunistic infections (paradoxical form) or the unmasking of previously subclinical, untreated infections (unmasking form) [10–12]. TB-associated IRIS has been reported in up to 43% of patients receiving concurrent treatment for these infections [13–28]. In an earlier publication where consensus case-definitions were applied retrospectively, 18 (7.5%) of 237 patients with TB at baseline had paradoxical TB-associated IRIS [29]. There has so far been only one [27] prospective study on TB-IRIS in India and most of the studies worldwide are also retrospective. In this communication, we report findings of a prospective study in HIV/AIDS patients started on highly active antiretroviral treatments (HAART) who were followed up at least for a predefined period for development of IRIS; TB-associated IRIS being of major interest.

## 2. Materials and Methods

We report findings of a prospective, observational study between November, 2008 to May, 2010 to determine the incidence of IRIS in patients with HIV-associated TB and to identify the risk factors for TB-IRIS. The study included adult HIV/AIDS patients who attended the outpatient department, ART clinic or were admitted to the All India Institute of Medical Sciences Hospital (AIIMS, New Delhi, India). The ART centre was opened at AIIMS hospital in May 2005 as part of National AIDS Control Programme started in April 2004. The hospital provides tertiary care to the population of Delhi and its neighbouring states and most of its patients come from the lower socioeconomic strata. The hospital and the clinic provides service to both newly diagnosed as well as referred cases. 

 Following were exclusion criteria: development of hypersensitivity to antiretroviral or antituberculosis drugs, past history of HAART, pregnancy, anticipated difficulty in followup and failure to give written informed consent. 

 Two hundred and twenty four HIV-infected ART-naïve adults who were recently diagnosed and started on HAART were divided into two groups: (i) HIV/AIDS patients with active TB and (ii) HIV/AIDS patients with no evidence of active TB at the time of recruitment in the study. HIV infection was documented by a licensed third generation ELISA kit as described in earlier study [30]. Tuberculosis was diagnosed as described in the previous study [31]. Culture and drug susceptibility testing (DST) for *Mycobacterium tuberculosis* were done in all cases where specimens were available to rule out drug-resistant TB. TB cases were divided into two categories—(i) definitive—where *Mycobacterium tuberculosis* was demonstrated in smear (Ziehl Neelsen method) and/or culture (Lowenstein Jensen) or *Mycobacterium tuberculosis*-polymerase chain reaction (*Mtb*-PCR) was positive in various body fluids (sputum, bronchoalveolar lavage fluid, pleural fluid, ascitic fluid, pericardial fluid, cerebrospinal fluid, bone marrow aspirate, pus specimens from cold abscesses) and (ii) probable—where specimen for smear and/or culture or *Mtb*-PCR was negative or could not be obtained due to technical difficulties. Criteria for diagnosis included: (a) pulmonary infiltrates located at classical site with no response to antibiotics; (b) exudative effusion or other body fluids with predominantly lymphocytes and elevated adenosine deaminase (ADA) activity (>35 U/L); (c) imaging (chest radiograph, ultrasonography, computed tomography and magnetic resonance imaging) highly suggestive of TB especially intrathoracic and abdominal lymph nodes with central hypodensity and peripheral rim enhancement; (d) imaging showing lesions in liver, spleen, intestine, bone and brain highly suggestive of TB (image-guided aspiration from lymph nodes or other lesions not possible due to technical difficulty); (e) clinical and radiological response to antiTB treatment during followup. Any one of criteria (a) to (d) along with (e) was required to be present for the diagnosis of probable TB. Both definitive and probable TB cases were included. The Institutional Ethics Committee approved the study. Written informed consent was obtained from all patients.

 Patients underwent a thorough clinical examination on enrolment and subsequently every month as they came to the ART clinic for followup and collection of antiretroviral drugs in accordance with the Guidelines of National AIDS Control Organisation (NACO) of Ministry of Health and Family Welfare, Government of India [32, 33]. All details were recorded in a predesigned proforma. Patients with TB were provided treatment free of cost from Directly Observed Treatment Short-Course (DOTS) centre in accordance with the Revised National Treatment Control Programme (RNTCP) of Ministry of Health and Family Welfare, Govt. of India [34, 35]. Anti-TB treatment was administered intermittently, thrice a week while the ART was administered daily as described earlier [34, 35]. The time of HAART initiation was decided as per NACO guidelines [32]. Medical social workers ensured regular visits of the patients to the DOTS and ART clinics.

 The minimum followup period was 3 months for development of IRIS. All the patients were followed up till the end of the study. Hence those who were recruited earlier would have had longer followup than those who were recruited later. Patients were contacted telephonically or their houses were visited in case they failed to turn up for their scheduled visits [33, 35]. End point of the study was development of TB-IRIS according to the case-definitions. Diagnosis of IRIS was based on published case-definitions criteria published by Meintjes et al. [36] for TB-associated IRIS and French et al. [37] for other cases. In cases where IRIS developed, change in CD4 count, plasma viral load, haematological and biochemical parameters were noted. CD4 count and HIV viral load were estimated using flow-cytometry (Becton Dickinson, USA) and Amplicor HIV-1 Monitor Test (Reverse Transcriptase Polymerase Chain Reaction, Roche Diagnostics Corp., Indianapolis), respectively.

### 2.1. Statistical Analysis

Continuous data are presented as mean ± standard deviation (for normally distributed variables) or median and interquartile range or IQR (for variable influenced by extreme values). Categorical data are presented as numbers with proportions, *n*(%). Percentage reduction in viral load has been calculated by dividing absolute reduction in viral load by baseline viral load and then multiplying it by 100. The categorical data were compared between groups by the Chi square or Fisher's exact test and continuous variables by the Mann-Whitney *U*-test. All tests were two-sided, and *P* < .05 was considered statistically significant. All analyses were using a statistical software package (Intercooled Stata 8.0 for Windows, Stata Corporation, College Station, TX, USA).

## 3. Results

**Figure 1:**
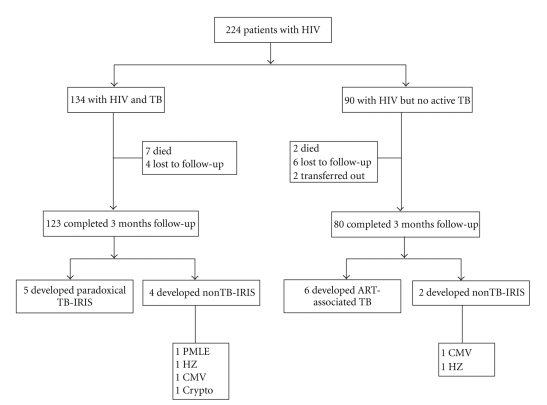
Study profile. HIV: human immunodeficiency virus; TB: tuberculosis; IRIS: immune reconstitution inflammatory syndrome; PMLE: progressive multifocal leukoencephalopathy; HZ: herpes zoster; CMV: cytomegalovirus; Crypto: cryptococcal meningitis.

Study data are summarized in Figure 1. As per case-definitions, all patients were followed-up for a minimum period of 3 months. A total of 203 patients (123 in HIV-TB group and 80 patients in HIV group) has completed 3 months followup and TB-IRIS data of these patients are being reported. 

 Pretreatment absolute CD4 count was significantly lower in HIV-TB group (137 (IQR 72–222) versus 198 (IQR 93–238) cells/*μ*l, *P* = .038) compared to HIV group (Table 1).

 HIV-TB group had 123 patients; 98 (80%) had extrapulmonary TB (EPTB) (39 (32%) of these had disseminated TB) while 25 (20%) patients had pulmonary TB (PTB) (Table 2). 

Forty eight of 123 (39%) patients had “definitive” TB and remaining 75 patients had “probable” TB. Of these 75 probable TB cases, all had imaging features compatible with TB, 38 of 75 patients had cytological and biochemical characteristics in body fluids (pleural effusion, ascites or CSF), 3 had histopathological findings highly suggestive of TB and all of them showed clinical and imaging response to antiTB treatment during followup (minimal followup 3 months in the last recruited patients). A detailed description has been provided in Tables 3a and 3b.

In total, 5 (4%) of 123 patients with TB at baseline started on HAART had paradoxical TB-associated IRIS (Table 4), while 6 (7.5%) of 80 patients without TB at baseline developed ART-associated TB after starting HAART. All 11 episodes of IRIS and ART-associated TB occurred within 3 months of starting HAART. Median intervals between initiation of HAART and occurrence of paradoxical TB-IRIS and ART-associated TB were 73 (IQR 6–84) and 37 (IQR 7–88) days, respectively. Comparisons of clinical characteristics of different groups of patients have been detailed in Tables 5, 6 and 7. None of these patients died during followup period. 

Most common clinical manifestation in patients with paradoxical worsening was new onset fever, anorexia and weight loss. A detailed description has been provided in Table 8. All had definitive TB (Table 9) and multidrug-resistant TB was ruled out by DST. All episodes were of mild to moderate severity not requiring any interruption of HAART. Hospitalization was required in 3 of 5 patients. Two patients required repeated aspiration of TB cervical cold abscesses. Symptoms of paradoxical deterioration resolved within one week with continued treatment, antipyretics (paracetamol), analgesics (nonsteroidal antiinflammatory drugs) and none required steroids.

 In 6 patients with ART-associated TB, 5 had pulmonary TB and 1 had TB lymphadenitis—diagnosis of TB was definitive in all cases (Tables 8, 9). None of these patients had an inflammatory clinical presentation, for example, TB lymphadenitis or abscesses with prominent acute inflammatory features or pulmonary tuberculosis complicated by respiratory failure due to adult respiratory distress syndrome, suggestive of unmasking TB-associated IRIS. None of these patients required hospitalization. All patients were administered antiTB treatment and showed good response to treatment. 

There were total 6 (2.7%) cases of nonTB-IRIS—2 patients with *herpes zoster*, 2 patients with *cytomegalovirus *infection (IgM positive and biopsy from gut lesion showing characteristic cytopathic effect), 1 patient with *cryptococcal *meningitis (India ink and *cryptococcal *antigen in CSF positive) and 1 patient with progressive multifocal leukoencephalopathy (confirmatory findings on MRI brain and culture for JC virus positive).

On univariate analyses, age, gender, body mass index, presence of extra-pulmonary/disseminated TB, baseline CD4 count, percentage change in CD4 and baseline viral load were not significantly associated with the development of paradoxical TB-associated IRIS or ART-associated TB. In patients who developed paradoxical TB-associated IRIS, the median interval between the initiation of antiTB treatment and HAART was shorter as compared to those who did not develop although the difference was not statistically significant (20 (IQR 18–27) versus 32 (IQR 21–70) days; *P* = .136). There was a significantly higher reduction in viral load (99.93 (IQR 99.91–99.97) versus 99.58 (IQR 98.85–99.9) %; *P* = .016) in patients who developed paradoxical TB-associated IRIS (Table 5). The similar trend was found in patients with ART-associated TB but it was not statistically significant (Table 6). However, when patients with any IRIS and ART-associated TB were compared with those who did not develop IRIS, statistically significant differences were found in increase in CD4 (136 (IQR 60–272) versus 53 (IQR 15–146) %; *P* = .023) and decrease in viral load (99.91 (IQR 99.8–99.93) versus 99.55 (IQR 98.76–99.9) %; *P* = .002). 

## 4. Discussion

In present study 4% of patients with HIV-associated TB developed paradoxical TB-associated IRIS after starting HAART and 7.5% of patients with no overt evidence of TB at initiation of HAART developed ART-associated TB. Only a few prospective studies have been published using the consensus case-definitions for TB-IRIS. Earlier studies from developed nations had reported a high incidence of TB-associated IRIS (17–43%) as compared to studies from developing nations (2–13%) [13–28]. Kumarasamy et al. [27] from southern India in their recent publication found the incidence of paradoxical TB-associated IRIS to be 5.5%. 

The results of present study are similar to a recent publication from our centre where authors applied consensus case-definitions criteria retrospectively [29] and in this study also no case of ART-associated TB fulfilling the criteria of unmasking TB-IRIS was reported. Incidence rates of paradoxical TB-IRIS and ART-associated TB were 7.5% and 3%, respectively in that study. Authors observed significant differences in baseline CD4 count and change in the CD4 cell count after 6 months between patients who developed paradoxical TB-IRIS and those who did not.

The higher incidence of TB-IRIS reported, particularly in the western literature, can be explained by leniency of clinical diagnostic criteria. Moreover, clinical diagnosis is readily made by the experts working in the field of HIV-TB. Hence, the staff of ART clinics should be adequately trained to suspect, categorise and confirm the diagnosis by appropriate investigations. Institutional adjudication committee may look into the diagnosis of IRIS. There should also be a proper reporting system to the higher agencies which could be further facilitated by establishing regional or national registry. An effort from the NACO in this regard will be highly welcome. The true incidence of TB-IRIS may be known from future studies with the application of consensus case definitions. 

The lack of application of uniform case-definitions for TB-associated IRIS across various published studies makes direct comparison of these results difficult. Recently, an International Network for the Study of HIV-associated IRIS (INSHI) case-definitions for TB-associated IRIS has been proposed for their use in resource-limited settings [36]. These have been validated in both retrospective and prospective studies [38, 39] and have shown good agreement with other published case definitions and expert opinions for IRIS. In the present study we have used these proposed case-definitions for the diagnosis of TB-associated IRIS.

Though the consensus case-definitions can be easily applied in resource-limited settings where it is not always possible to get CD4 count or plasma viral load readily and regularly, it seems to have some limitations. First, it would have missed the patients who developed paradoxical TB-associated IRIS after 90 days of HAART—while it is still a possibility, even keeping in mind the chance of a new infection. Second, the definition for unmasking TB-IRIS seems to be too stringent to be applicable in clinical practice and the arbitrary cut-off of 3 months is likely to miss a few cases. 

Though none of ART-associated TB met the criteria of heightened inflammatory response to be categorised as unmasking TB-IRIS in both studies from our centre, clinicians should remain highly vigilant in HIV patients who develop TB after starting ART, as they can have much more severe disease and can deteriorate rapidly due to immune reconstitution and may even require hospitalization.

In the present study no statistically significant influence of age, gender, BMI, baseline CD4 or viral load and magnitude of change in CD4 were found on occurrence of TB-IRIS as opposed to the findings of previous studies [13–28]. Early initiation of HAART after antiTB treatment was associated with paradoxical TB-associated IRIS though not statistically significant as mentioned in previous studies [13–28]. But decrease in viral load was associated with paradoxical TB-IRIS as proposed in the literature [4–8]. Extra-pulmonary and disseminated TB has been reported to be associated with the occurrence of TB-associated IRIS in previous studies. Though in our study 4 out of 5 paradoxical reactions occurred in patients with extra-pulmonary TB and only 1 of those had disseminated TB. These discrepancies could possibly be due to the small number of IRIS events in the present study. Though the patients were explained thoroughly about the possible symptoms of IRIS and strictly instructed to visit the clinic or contact the investigator on occurrence of any symptoms suspicious of IRIS, monthly followup schedule might have missed a few cases of IRIS occurring in between. Another possible explanation for low incidence of IRIS could be the use of intermittent thrice weekly antiTB treatment in place of daily drug regimen. 

In view of relatively low incidence of TB-IRIS in South-East Asia, the small sample size of the present study makes it very difficult to identify the risk factors associated with it. 

Extrapulmonary TB which is much more common in advanced cases of HIV/AIDS has a very low bacillary load in samples and it is sometimes not possible to get specimens for microbiological or histopathological diagnosis due to difficulties with imaging guided procedures [40, 41]. Moreover, in the developing nations with resource-limited settings, as opposed to the present study which was conducted in a tertiary care centre, state-of-the-art imaging for both diagnosis and followup and other diagnostic facilities (like ADA level estimation, PCR) are not available in peripheral centres. The same holds true for diagnosis of other opportunistic infections particularly viral, fungal and protozoal infestations. Availability of limited laboratory facilities in the public hospitals, use of commercial laboratories by the patients and a lack of accreditation and quality assurance of these laboratories are real problems. However, it is to be emphasized here that these definitions have been formulated for use in the resource-limited settings like ours where access to such investigations is expected to be limited.

We did not encounter any death due to IRIS. But it will be prudent to keep in mind that IRIS affecting central nervous system or producing acute respiratory distress syndrome can be lethal if not diagnosed timely and treated appropriately. Since, till date no single clinical factor has been identified which can predict development of IRIS, a high index of clinical suspicion is still the key for an early diagnosis of IRIS. Further research is needed to better understand the immune-pathogenesis of the various types of IRIS so that better diagnostic tests and effective therapies can be developed.

## 5. Conclusion

TB-associated IRIS reactions are important and may complicate management of HIV-TB coinfection. They should be promptly recognised and treated. Most cases of TB-associated IRIS can be managed easily and do not need discontinuation of ART. The consensus case-definitions seem to be a useful tool in the diagnosis of TB-associated IRIS in resource-poor countries where access to laboratory facilities is limited.

## Figures and Tables

**Table 1:** Baseline characteristics of 203 patients with HIV/AIDS.

	HIV-TB (*n* = 123)	HIV (*n* = 80)
Age (yrs)	36 ± 10	35 ± 9
Sex (% male)	80*	59*
BMI (kg/m^2^)	19 ± 4.5	19.6 ± 3.8
CD4 (cells/*μ*l)	137 (72–222)^†^	198 (93–238)^†^
Plasma VL (log_10_ copies/ml)	5.23 (4.79–5.65)	5.09 (4.55–5.65)

BMI: body mass index; VL: viral load

Age and BMI are presented as mean ± SD; gender as % of male and CD4 cell counts and viral load as median and IQR

*P = .001

^†^ *P* = .038.

**Table 2:** Detailed description of 123 TB patients in the HIV-TB group.

Type/site of TB	HIV-TB group
PTB [*n*(%)]	25 (20)*
EPTB [*n*(%)]	98 (80)
DTB [*n*(%)]	39 (32)
Lung + Pleura + mediastinal LN [*n*(%)]	11 (9)*
Pleura + RP/mesenteric LN [*n*(%)]	6 (5)
Lung + pleura [*n*(%)]	4 (3)
Ascites + RP/mesenteric LN + mediastinal LN [*n*(%)]	4 (3)
Ascites + hepatosplenomegaly + RP/mesenteric LN + mediastinal LN [*n*(%)]	3 (2)
Lung + pleura + Ascites + RP/mesenteric LN [*n*(%)]	3 (2)
Ascites + hepatosplenomegaly + mediastinal LN [*n*(%)]	2 (2)
Lung + pleura + Ascites + hepatosplenomegaly + RP/mesenteric LN [*n*(%)]	2 (2)
Lung + pleura + Ileocaecal + RP/mesenteric LN [*n*(%)]	2 (2)
Cervical LN + mediastinal LN + RP/mesenteric LN [*n*(%)]	2 (2)
Abdomen [*n*(%)]	23 (19)
Ascites + RP/mesenteric LN [*n*(%)]	8 (7)
Ascites + hepatosplenomegaly + RP/mesenteric LN [*n*(%)]	7 (6)
Ascites + hepatosplenomegaly [*n*(%)]	4 (3)
Ileocaecal + RP/mesenteric LN [*n*(%)]	4 (3)
Lymph node [*n*(%)]	16 (13)
Cervical [*n*(%)]	10 (8)^†^
Abdominal [*n*(%)]	4 (3)
RP + mesenteric LN	2 (2)
RP LN	1 (1)
Periportal + mesenteric LN	1 (1)
Mediastinal [*n*(%)]	2 (2)
Pleural effusion [*n*(%)]	11 (9)
CNS [*n*(%)]	6 (5)
Meningitis [*n*(%)]	3 (2)
Tuberculoma [*n*(%)]	2 (2)
Meningitis + Tuberculoma [*n*(%)]	1 (1)
MTB [*n*(%)]	2 (2)
Pott's spine [*n*(%)]	1 (1)

PTB: pulmonary tuberculosis; EPTB: extra pulmonary tuberculosis; RP: retroperitoneal; LN: lymph nodes; CNS: central nervous system; DTB: disseminated tuberculosis; MTB: military tuberculosis

EPTB is presented as % of TB; Types of EPTB as % of EPTB; Subtypes of EPTB types as % of EPTB along with absolute no. of cases

*One patient each from these groups developed paradoxical TB-IRIS

^†^Three patients from this group developed paradoxical TB-IRIS.

**Table:** (a)

Specimen type	Test (positive/performed)			
	Smear	Culture	TB-PCR	Overall
Sputum	12^‡^/25	—	—	12
BAL	0/4	2/4	—	2
Pleural fluid	0/39	0/39	9/39	9
Ascitic fluid	0/28	0/28	6/28	6
CSF	0/6	2/6	3/6	3
Bone marrow aspirate	0/2	2/2	2/2	2
LN aspirate	14^§^/27^†^	2/2	2/2	14

Total				48

**Table:** (b)

Characteristic findings	No. present
(i) Radiological^†^	75^¶,*||*,^**
(ii) Body fluids^‡^	39
Pleural effusion	22^¶^
Ascites	14
CSF	3
(iii) Histopathological^§^	3^*||*^
(i) + (ii) + (iii)	1
(i) + (ii)	38^¶^
(i) + (iii)	2^*||*^
(i)	34**

Total	75

**Table 4:** Diagnosis of paradoxical TB-IRIS according to consensus case-definition.

Sl. no.	TB		TB-IRIS	Clinical criteria*	
	Site	Category		Major	Minor
(1)	DTB (Lung + Pleura + mediastinal LN)	Probable	DTB (Lung + Pleura + mediastinal + RP/mesenteric LN)	New LN	Worsening constitutional symptoms (fever, weight loss, etc.)

(2)	LNTB (cervical)	Definitive	DTB (cervical + mediastinal + RP/mesenteric LN)	New LN	Worsening constitutional symptoms

(3)	LNTB (cervical)	Probable	DTB (cervical + mediastinal + RP/mesenteric LN)	New LN	Worsening constitutional symptoms

(4)	PTB	Definitive	PTB	Worsening CXR	Worsening constitutional symptoms Worsening respiratory symptoms (cough, dyspnoea, etc.)

(5)	LNTB (cervical)	Probable	LNTB (cervical)	New & enlarging LN	Worsening constitutional symptoms

PTB: pulmonary tuberculosis; RP: retroperitoneal; LN: lymph nodes; DTB: disseminated tuberculosis

*“Antecedent requirements” and “Alternative explanations for clinical deterioration” criteria were fulfilled in all the cases.

**Table 5:** Comparison characteristics between patients with and without TB-IRIS in 123 patients with HIV-TB.

	No. TB-IRIS	TB-IRIS
	(*n* = 118)	(*n* = 5)
Age (yrs)	37 ± 10	31 ± 7
Sex (% male)	80	80
BMI (kg/m^2^)	19 ± 5	18 ± 3
ATT-ART (days)	32 (21–70)	20 (18–27)
CD4 (cells/*μ*l)	136 (70–225.75)	167 (118–193)
Increase in CD4	89 (32.25–144.75)	229 (61–283)
(cells/*μ*l)		
Increase in CD4 (%)	57.6 (24–181)	119 (63.5–207)
VL (log_10_ copies/ml)	5.21 (4.76–5.65)	5.49 (5.18–5.63)
Decrease in VL	5.19 (4.76–5.63)	5.49 (5.18–5.63)
(log_10_ copies/ml)		
Decrease in VL (%)	99.58 (98.85–99.9)*	99.93 (99.91–99.97)*

BMI: body mass index; ATT: antituberculosis treatment; ART: antiretroviral treatment; VL: viral load

Age and BMI are presented as mean ± SD; gender as % of male; EPTB as % of TB; DTB as % of EPTB and ATT-ART gap, CD4 cell counts and viral load as median and IQR

*P = .016.

**Table 6:** Comparison of characteristics between patients with and without ART-associated TB in 80 patients with HIV.

	No TB	ART-associated TB
	(*n* = 74)	(*n* = 6)
Age (yrs)	35 ± 9	35 ± 15
Sex (% male)	60	50
BMI (kg/m^2^)	19.7 ±3.8	19.4 ± 4
CD4 (cells/*μ*l)	200 (94.25–242)	132 (57.25–182.75)
Increase in CD4	88 (27.25–149.75)	161 (129–316)
(cells/*μ*l)		
Increase in CD4 (%)	46 (11.5–126.5)	160 (72–369)
VL (log_10_ copies/ml)	5.09 (4.54–5.65)	5.28 (4.95–5.51)
Decrease in VL	5.08 (4.5–5.57)	5.13 (4.87–5.39)
(log_10_ copies/ml)		
Decrease in VL (%)	99.58 (98.79–99.9)	99.91 (99.84–99.93)

BMI: body mass index; VL: viral load

Age and BMI are presented as mean ± SD; gender as % of male and CD4 cell counts and viral load as median and IQR

There was no statistically significant difference.

**Table 7:** Comparison of characteristics between HIV-TB patients without and with TB-IRIS and HIV patients with ART associated TB.

	No TB-IRIS	TB-IRIS	ART-associated TB
	(*n* = 118)	(*n* = 5)	(*n* = 6)
Age (yrs)	37 ± 10	31 ± 7	35 ± 15
Sex (% male)	80	80	50
BMI (kg/m^2^)	19 ± 5	18 ± 3	19.4 ± 4
ATT-ART (days)	32 (21–70)	20 (18–27)	—
ART-IRIS (days)	—	73 (6–84)	37 (7–88)
CD4 (cells/*μ*l)	136 (70–225.75)	167 (118–193)	132 (57.25–182.75)
Increase in CD4 (cells/*μ*l)	89 (32.25–144.75)	229 (61–283)	161 (129–316)
Increase in CD4 (%)	57.6 (24–181)	119 (63.5–207)	160 (72–369)
VL (log_10_ copies/ml)	5.21 (4.76–5.65)	5.49 (5.18–5.63)	5.28 (4.95–5.51)
Decrease in VL (log_10_ copies/ml)	5.19 (4.76–5.63)	5.49 (5.18–5.63)	5.13 (4.87–5.39)
Decrease in VL (%)	99.58 (98.85–99.9)	99.93 (99.91–99.97)	99.91 (99.84–99.93)

BMI: body mass index; ATT: antituberculosis treatment; ART: antiretroviral treatment; VL: viral load

Age and BMI are presented as mean ± SD; gender as % of male and ATT-ART gap, ART-IRIS gap, CD4 cell counts and viral load as median and IQR.

**Table 8:** Description of TB in patients with TB-IRIS (at the time of IRIS) and ART-associated TB.

	Paradoxical TB-IRIS (*n* = 5)*	ART-associated TB (*n* = 6)
PTB [*n* (%)]	1	5
EPTB [*n* (%)]	4	1
DTB [*n* (%)]	3	—
Lung + Pleura + mediastinal LN + RP/mesenteric LN [*n* (%)]	1	—
Cervical LN + mediastinal LN + RP/mesenteric LN [*n* (%)]	2	—
Lymph node [*n* (%)]	1	1
Cervical [*n* (%)]	1	1

PTB: pulmonary tuberculosis; EPTB: extrapulmonary tuberculosis; DTB: disseminated tuberculosis; LN: lymph node

EPTB, types of EPTB and subtypes of EPTB types presented in absolute no. of cases

*Three of the 5 patients with paradoxical IRIS had cervical TB lymphadenopathy at presentation while one had disseminated and the other had pulmonary TB. After being started on ART, one with cervical TB lymphadenopathy developed enlargement of the previously existing lymph nodes along with appearance of new nodes in the neck; other two with cervical TB lymphadenopathy developed disseminated TB; in one with disseminated TB (lung, pleura and mediastinum) the disease became more extensive (with abdominal involvement); and one with pulmonary TB developed new chest infiltrates with increased bacillary load in the sputum.

**Table 9:** Method of TB diagnosis in patients with TB-IRIS (at the time of IRIS) and ART-associated TB.

Specimen type	Test (positive/performed)			
	Smear	Culture	TB-PCR	Overall
TB-IRIS				

Sputum	1/1	1/1	—	1
LN aspirate	4/4	4/4	4/4	4

Total				5

ART-associated TB				

Sputum	4/5	5/5	—	5
LN aspirate*	1/1	—	—	1

Total				6

PCR: polymerase chain reaction; LN: lymph node

*Fine needle aspiration was done in this case and the specimen also showed necrotizing granulomas on histopathological examination.

## References

[1] Corbett EL, Watt CJ, Walker N (2003). The growing burden of tuberculosis: global trends and interactions with the HIV epidemic. *Archives of Internal Medicine*.

[2] Wood R, Maartens G, Lombard CJ (2000). Risk factors for developing tuberculosis in HIV-1-infected adults from communities with a low or very high incidence of tuberculosis. *Journal of Acquired Immune Deficiency Syndromes and Human Retrovirology*.

[3] Keiser O, Anastos K, Schechter M (2008). Antiretroviral therapy in resource-limited settings 1996 to 2006: patient characteristics, treatment regimens and monitoring in sub-Saharan Africa, Asia and Latin America. *Tropical Medicine and International Health*.

[4] Battegay M, Nüesch R, Hirschel B, Kaufmann GR (2006). Immunological recovery and antiretroviral therapy in HIV-1 infection. *Lancet Infectious Diseases*.

[5] Dean GL, Edwards SG, Ives NJ (2002). Treatment of tuberculosis in HIV-infected persons in the era of highly active antiretroviral therapy. *AIDS*.

[6] De Jong BC, Israelski DM, Corbett EL, Small PM (2004). Clinical management of tuberculosis in the context of HIV infection. *Annual Review of Medicine*.

[7] Havlir DV, Barnes PF (1999). Tuberculosis in patients with human immunodeficiency virus infection. *New England Journal of Medicine*.

[8] Burman WJ, Jones BE (2001). Treatment of HIV-related tuberculosis in the era of effective antiretroviral therapy. *American Journal of Respiratory and Critical Care Medicine*.

[9] French MA (2009). Immune reconstitution inflammatory syndrome: a reappraisal. *Clinical Infectious Diseases*.

[10] Lawn SD, Bekker L-G, Miller RF (2005). Immune reconstitution disease associated with mycobacterial infections in HIV-infected individuals receiving antiretrovirals. *Lancet Infectious Diseases*.

[11] Shelburne SA, Hamill RJ, Rodriguez-Barradas MC (2002). Immune reconstitution inflammatory syndrome: emergence of a unique syndrome during highly active antiretroviral therapy. *Medicine*.

[12] French MA, Price P, Stone SF (2004). Immune restoration disease after antiretroviral therapy. *AIDS*.

[13] Narita M, Ashkin D, Hollender ES, Pitchenik AE (1998). Paradoxical worsening of tuberculosis following antiretroviral therapy in patients with aids. *American Journal of Respiratory and Critical Care Medicine*.

[14] Wendel KA, Alwood KS, Gachuhi R, Chaisson RE, Bishai WR, Sterling TR (2001). Paradoxical worsening of tuberculosis in HIV-infected persons. *Chest*.

[15] Navas E, Martín-Dávila P, Moreno L (2002). Paradoxical reactions of tuberculosis in patients with the acquired immunodeficiency syndrome who are treated with highly active antiretroviral therapy. *Archives of Internal Medicine*.

[16] Breton G, Duval X, Estellat C (2004). Determinants of immune reconstitution inflammatory syndrome in HIV type 1-infected patients with tuberculosis after initiation of antiretroviral therapy. *Clinical Infectious Diseases*.

[17] Kumarasamy N, Chaguturu S, Mayer KH (2004). Incidence of immune reconstitution syndrome in HIV/tuberculosis-coinfected patients after initiation of generic antiretroviral therapy in India. *Journal of Acquired Immune Deficiency Syndromes*.

[18] Michailidis C, Pozniak AL, Mandalia S, Basnayake S, Nelson MR, Gazzard BG (2005). Clinical characteristics of IRIS syndrome in patients with HIV and tuberculosis. *Antiviral Therapy*.

[19] Bourgarit A, Carcelain G, Martinez V (2006). Explosion of tuberculin-specific Th1-responses induces immune restoration syndrome in tuberculosis and HIV co-infected patients. *AIDS*.

[20] Chew NS, Brannigan E, Nugent C Immune reconstitution inflammatory syndrome of tuberculosis among HIV-infected patients receiving antituberculous and antiretroviral therapy in a north Dublin inner city hospital.

[21] Manosuthi W, Kiertiburanakul S, Phoorisri T, Sungkanuparph S (2006). Immune reconstitution inflammatory syndrome of tuberculosis among HIV-infected patients receiving antituberculous and antiretroviral therapy. *Journal of Infection*.

[22] Elliott JH, Sarun S, Mean CV Tuberculosis-associated immune restoration disease is associated with increased PPD-specific T cell responses detected by a whole blood interferon-*γ* release assay.

[23] Lawn SD, Myer L, Bekker L-G, Wood R (2007). Tuberculosis-associated immune reconstitution disease: incidence, risk factors and impact in an antiretroviral treatment service in South Africa. *AIDS*.

[24] Park WB, Choe PG, Jo JH (2007). Tuberculosis manifested by immune reconstitution inflammatory syndrome during HAART. *AIDS*.

[25] Serra FC, Hadad D, Orofino RL (2007). Immune reconstitution syndrome in patients treated for HIV and tuberculosis in Rio de Janeiro. *Brazilian Journal of Infectious Diseases*.

[26] Eshun-Wilson I, Havers F, Nachega J Evaluating TB-associated immune reconstitution infl ammatory syndrome using standardized case definitions.

[27] Kumarasamy N, Venkatesh K, Vignesh V Immunologic outcome following HAART among HIV-infected patients developing immune reconstitution inflammatory syndrome of tuberculosis in South India.

[28] Manosuthi W, Van Tieu H, Mankatitham W Clinical case definition and manifestations of paradoxical tuberculosis (TB) immune reconstitution inflammatory syndrome (IRIS).

[29] Sharma SK, Dhooria S, Barwad P (2010). A study of TB-associated immune reconstitution inflammatory syndrome using the consensus case-definition. *Indian Journal of Medical Research*.

[30] Sharma SK, Saha PK, Dixit Y, Siddaramaiah NH, Seth P, Pande JN (2000). HIV seropositivity among adult tuberculosis patients in Delhi. *The Indian Journal of Chest Diseases &amp; Allied Sciences*.

[31] Sharma S, Rathored J, Ghosh B, Sharma SK (2010). Genetic polymorphisms in TNF genes and tuberculosis in North Indians. *BMC Infectious Diseases*.

[32] National AIDS Control Organisation (NACO) (2004). *National Guidelines for Implementation of Antiretroviral Therapy*.

[33] Sharma SK, Dhooria S, Prasad KT (2010). Outcomes of antiretroviral therapy in a northern Indian urban clinic. *Bulletin of the World Health Organization*.

[34] Sharma SK, Lawaniya S, Lal H, Singh UB, Sinha PK (2004). DOTS centre at a tertiary care teaching hospital: lessons learned and future directions. *The Indian Journal of Chest Diseases &amp; Allied Sciences*.

[35] Tahir M, Sharma SK, Rohrberg D-S, Gupta D, Singh UB, Sinha PK (2006). DOTS at a tertiary care center in northern India: successes, challenges & the next steps in tuberculosis control. *Indian Journal of Medical Research*.

[36] Meintjes G, Lawn SD, Scano F (2008). Tuberculosis-associated immune reconstitution inflammatory syndrome: case definitions for use in resource-limited settings. *The Lancet Infectious Diseases*.

[37] French MA, Price P, Stone SF (2004). Immune restoration disease after antiretroviral therapy. *AIDS*.

[38] Eshun-Wilson I, Havers F, Nachega JB (2010). Evaluation of paradoxical TB-associated IRIS with the use of standardized case definitions for resource-limited settings. *Journal of the International Association of Physicians in AIDS Care*.

[39] Haddow LJ, Moosa M-YS, Easterbrook PJ (2010). Validation of a published case definition for tuberculosis-associated immune reconstitution inflammatory syndrome. *AIDS*.

[40] Brodie D, Schluger NW (2005). The diagnosis of tuberculosis. *Clinics in Chest Medicine*.

[41] Sharma SK, Mohan A (2004). Extrapulmonary tuberculosis. *Indian Journal of Medical Research*.

